# Insight into Molecular Mechanisms and Pathways Related to the Association of Periodontal Disease and Type 2 Diabetes Mellitus

**DOI:** 10.2174/0118715303415067251124044844

**Published:** 2026-01-12

**Authors:** Hanim Afzan Ibrahim, Wan Majdiah Wan Mohamad, Noraini Mohamad, Nur Karyatee Kassim, Tuan Nadrah Naim Tuan Ismail, Wan Suriana Wan Ab Rahman, Zainab Mat Yudin, Srijit Das, Siti Rosmani Md Zin

**Affiliations:** 1 Medical and Basic Dental Sciences Unit, School of Dental Sciences, Health Campus, Universiti Sains Malaysia, Kubang Kerian, Kota Bharu 16150, Kelantan, Malaysia;; 2 College of Medicine & Health Sciences, Sultan Qaboos University, Sultanate of Oman, Muscat, Oman

**Keywords:** Periodontal disease, gingivitis, periodontitis, type 2 diabetes, insulin resistance, systemic inflammation

## Abstract

**Introduction:**

Periodontal disease is a condition that damages the supporting tissues, potentially resulting in tooth extraction, while type 2 diabetes is a condition that involves insulin resistance, leading to hyperglycaemia and systemic inflammation.

**Methods:**

A broad literature search was conducted in PubMed, Scopus, Web of Science, Google Scholar, and ProQuest. Search terms included combinations of keywords related to periodontal disease, type 2 diabetes, bone metabolism, genetics/epigenetics, inflammation, and oxidative stress, refined using Boolean operators. Titles and abstracts were screened, and eligible full-text articles were reviewed for relevant data.

**Results and Discusion:**

This review found that periodontal disease, a major cause of tooth loss in adults, is strongly influenced by the bidirectional relationship with type 2 diabetes. Hyperglycaemia in poorly controlled diabetes exacerbates periodontal inflammation by enhancing the formation of advanced glycation end-products, triggering pro-inflammatory pathways such as the activation of Nuclear Factor kappa-light-chain-enhancer of activated B cells and cytokine release (*e.g.*, Tumour Necrosis Factor-α, Interleukin [IL]-6, IL-18). Concurrent dysbiosis of the oral microbiome disrupts immunoregulation, while an imbalance in the receptor activator of nuclear factor kappa-B ligand (RANKL) and osteoprotegerin (OPG) system promotes osteoclastogenesis, collectively leading to accelerated tissue destruction, impaired healing, and an increased risk of complications.

**Conclusion:**

This review clarifies the molecular mechanisms of the triad axis of oral microbiota-inflammatory factors-bone metabolism markers in the bidirectional association between periodontal disease and type 2 diabetes. Understanding the underlying mechanisms of this bidirectional relationship can provide valuable information for researchers to identify potential targets for effective management strategies for periodontal disease in patients with type 2 diabetes.

## 1 INTRODUCTION

### 1.1 Periodontal Disease

Periodontal disease, commonly referred to as gum disease, is a severe infection affecting the gums and causing damage to the delicate tissues surrounding the teeth. This structure comprises the gingival tissue, alveolar bone, cementum, and periodontal ligament. Periodontitis represents the advancement of the periodontal condition from gingivitis to a persistent, deleterious, and irreversible inflammatory disease. Subsequently, bacteria gain access to deeper tissue layers and the surrounding periodontium, provoking a defensive reaction from the host to counter the invading pathogens [1].

The updated framework of the periodontal classification system, introduced by the American Academy of Periodontology (AAP) in 2017, is designed to offer a more thorough and precise structure for diagnosing and treating periodontal and peri-implant diseases and conditions. This revised classification divides periodontitis into three categories: periodontitis, necrotising periodontal diseases, and periodontitis as a manifestation of systemic diseases [1].

Periodontal disease affects about 90% of the global population, making it the most widespread oral condition. In the United States, roughly 50% of adults have some form of gingivitis, while up to 80% have experienced some form of periodontal disease during their lifetime [2]. In Malaysia, 94% of adults aged 15 years and above are diagnosed with periodontal disease, with 48.5% of them having periodontitis [3].

In the absence of intervention, periodontitis has the potential to erode the alveolar bone supporting the teeth in a continuous, irreversible process, resulting in tooth instability or eventual tooth loss [1, 4]. Periodontal disease can be treated with non-surgical or surgical methods. The non-surgical approach involves mechanical debridement of the tooth surface with curettes, ultrasonic devices, or lasers, alone or in combination with local antibiotics or antiseptics [4]. Meticulous oral hygiene by patients is a routine practice, and the use of interdental brushes and dental floss in areas that are difficult to access is essential. However, mechanical non-surgical treatment alone is usually ineffective in most advanced periodontitis lesions; therefore, a surgical approach is recommended in such cases [4].

### 1.2 Diabetes Mellitus

Diabetes mellitus is a chronic metabolic disorder characterised by elevated blood glucose levels due to abnormalities in insulin secretion, action, or both [5]. It encompasses a group of metabolic disorders in which glucose is inadequately utilised for energy and excessively produced due to abnormal gluconeogenesis and glycogenolysis, resulting in hyperglycaemia [6]. Diagnosis involves demonstrating elevated glucose concentrations in venous plasma or increased glycated haemoglobin (HbA1c) levels in the blood [7, 8].

Diabetes is classified into several categories, namely type 1 or type 2 diabetes, gestational diabetes mellitus, and other specific types based on causes such as genetic factors, pancreatic disorders, or medications [7, 8]. Type 1 diabetes (T1D) arises from autoimmune beta (β)-cell destruction, usually leading to absolute insulin deficiency, and includes latent autoimmune diabetes in adults. Type 2 diabetes occurs when the β-cells gradually lose their ability to produce sufficient insulin, often in the presence of insulin resistance and metabolic syndrome. Gestational diabetes mellitus is diabetes first diagnosed in the second or third trimester of pregnancy in women who did not have clear diabetes prior to pregnancy. It excludes other types of diabetes, such as T1D, that may occur during pregnancy. Specific types of diabetes include neonatal diabetes, maturity-onset diabetes of the young, cystic fibrosis–related diabetes, pancreatitis-related diabetes, and drug- or chemical-induced diabetes caused by glucocorticoids, HIV treatments, or organ transplantation [9].

Diabetes mellitus represents a widespread global public health concern, placing a substantial burden on healthcare systems and socioeconomic progress worldwide [10]. Globally, diabetes prevalence among adults was 8.8% in 2017 and is projected to rise to 9.9% by 2045 [11]. According to the most recent 10th edition of the International Diabetes Federation (IDF) Diabetes Atlas (2021), 537 million adults aged 20 to 79 years are diagnosed with diabetes worldwide [12].

In Malaysia, the 2023 National Health and Morbidity Survey (NHMS) reported a diabetes mellitus prevalence of 15.6% among adults [13, 14]. According to the IDF, the prevalence of diabetes reached 20%, affecting almost 4.43 million Malaysian adults in 2020 [12, 15].

Chronic hyperglycaemia in diabetes can cause long-term damage to various organs, including the eyes, kidneys [16], heart, nerves, and blood vessels [8]. Such damage leads to microvascular and macrovascular complications, which significantly contribute to morbidity and mortality [5]. A recent review found that in newly diagnosed type 2 diabetes in low- and middle-income countries, the prevalence of microvascular complications was 12% for retinopathy, 15% for nephropathy, and 16% for neuropathy. For macrovascular complications, the prevalence was 10% for ischaemic heart disease, 6% for peripheral arterial disease, and 2% for stroke [17]. Moreover, type 2 diabetes is a major factor in the development of diabetic nephropathy, which affects the kidneys [16].

This highlights the serious health impacts of diabetes, which also shares a reciprocal detrimental relationship with periodontal disease. Diabetes is a major risk factor for periodontal disease, and individuals with both conditions may experience worsened glycaemic control and increased risks of complications such as end-stage renal disease and cardiovascular disease [18]. Furthermore, type 2 diabetes significantly increases the susceptibility to and severity of periodontal diseases [19]. Animal studies have demonstrated adverse effects of diabetes on multiple cell types in periodontal tissue, including leukocytes, mesenchymal stem cells, vascular cells, periodontal ligament fibroblasts, osteoblasts, and osteocytes [19]. Diabetes mellitus can also impair new bone formation in response to bacterial influence [19].

This review provides a fundamental understanding of the role of oral microbiota, inflammatory mediators, and bone metabolism markers in the bidirectional association between periodontal disease and type 2 diabetes. It also considers related aspects, including microbiome–immune–metabolic interactions, tooth loss as a major clinical outcome of advanced periodontitis, and the role of genes in the predisposition and progression of periodontitis and type 2 diabetes. By outlining the key biological interactions, this review aims to clarify how these conditions are interconnected and to highlight potential targets for the development of effective prevention and management strategies for periodontal disease in patients with type 2 diabetes.

## 2 METHODOLOGY

A narrative literature search was conducted to explore the association between periodontal disease and type 2 diabetes, covering publications from 1997 to 2024. The search was performed using the following databases: PubMed, Scopus, Web of Science, Google Scholar, and ProQuest. Keywords used in the search included combinations of “periodontal disease”, “periodontitis”, “type 2 diabetes”, “type 2 diabetes mellitus”, “insulin resistance”, “bone metabolism marker”, “tooth extraction”, “genes”, “genetics”, “epigenetic”, “inflammatory markers”, “oxidative stress markers”, and “tooth extraction” using appropriate Boolean operators (AND, OR) to refine the results. Studies were included if they focused on the association between periodontal disease and type 2 diabetes, were published in English, involved either human or animal subjects, and were available in abstract or full text. Exclusion criteria included articles unrelated to the topic, those focusing exclusively on type 1 diabetes or other types of diabetes, non-English publications, and non-original research such as editorials, commentaries, and conference abstracts.

## 3 BIDIRECTIONAL ASSOCIATION OF PERIODONTAL DISEASE AND TYPE 2 DIABETES

The complex bidirectional relationship between periodontal disease and type 2 diabetes involves a network of biological and environmental risk factors, including insulin resistance and tobacco smoking. Dysbiosis of the oral microbiome and disruptions in immune–metabolic pathways also play pivotal roles in disease pathogenesis. These interactions influence clinical outcomes such as tooth extraction, in which oxidative stress, inflammatory mediators, and bone metabolism markers critically affect tissue healing and disease progression (Table **1**). The following subsections explore these mechanisms to elucidate the multifactorial nature of the association between periodontal disease and type 2 diabetes.

### 3.1 Insulin Resistance in Type 2 Diabetes

Type 2 diabetes results from insulin resistance and defective insulin secretion by pancreatic β-cells [20, 21]. Normally, insulin acts on classical target tissues—liver, adipose tissue, and muscle—by binding to the insulin tyrosine kinase receptor. This receptor phosphorylates insulin receptor substrate-1 (IRS-1), which subsequently activates phosphoinositide 3-kinase (PI3-kinase). PI3-kinase then phosphorylates phosphatidylinositol 4,5-bisphosphate (PIP2), leading to activation of Akt/Protein kinase B (PKB) [22]. This activation results in glucose transporter 4 (GLUT4) translocation from intracellular vesicles to the plasma membranes of adipocytes and skeletal muscle cells. Consequently, these cells can absorb extracellular glucose, thereby reducing interstitial glucose levels and ultimately lowering plasma glucose concentration [22].

However, in type 2 diabetes, insulin-sensitive tissues fail to respond appropriately to insulin, resulting in persistently high blood glucose, a condition known as insulin resistance. Elevated glucose levels trigger higher expression of lipopolysaccharide (LPS)-induced interleukin (IL)-6 mediated by GLUT4 [23]. Studies in animals and humans show that this metabolic disorder enhances inflammatory events in periodontal tissue, as indicated by increased levels of systemic inflammatory markers [19, 24, 25].

### 3.2 Tobacco Smoking

Tobacco smoking is widely recognised as a major public health concern, linked to numerous chronic diseases, including diabetes and periodontal disease. Both conditions are characterised by chronic inflammation and share a bidirectional relationship, in which one can exacerbate the other. Smoking not only increases the risk of developing diabetes and periodontitis individually but also intensifies their connection by amplifying molecular mechanisms that promote inflammation and tissue destruction.

In diabetic individuals, high blood sugar leads to the formation of advanced glycation end products (AGEs), which accumulate in periodontal tissues. AGEs bind to their receptors (RAGE) on periodontal cells, activating nuclear factor-kappa B (NF-κB) and promoting the release of pro-inflammatory cytokines [26]. Smoking exacerbates this process by increasing oxidative stress and promoting the accumulation of AGEs, thereby amplifying inflammation in both periodontal and systemic tissues. This enhanced inflammatory response further impairs glycaemic control in diabetic patients, creating a vicious cycle that perpetuates both diabetes and periodontitis. Patients with periodontitis and type 2 diabetes show markedly higher levels of AGEs in gingival crevicular fluid (GCF) compared to individuals with normal blood sugar and no periodontitis, highlighting the harmful effect of diabetes on periodontal health [27].

The impaired wound healing observed in diabetic patients is further compounded by smoking. Smoking reduces blood flow to periodontal tissues due to nicotine-induced vasoconstriction, limiting the delivery of oxygen and nutrients necessary for tissue repair. Moreover, the upregulation of matrix metalloproteinases (MMPs) in response to smoking accelerates the degradation of collagen and other extracellular matrix components, hindering tissue regeneration [28, 29]. This impaired healing response in the periodontal tissues of diabetic smokers may lead to the persistence and progression of periodontitis.

However, definitive mechanisms by which smoking affects diabetic smokers remain unclear. Additionally, a Korean study suggested that smoking significantly impacts the periodontal health of Korean adults, but the combined effect of smoking and diabetes was not significant [30].

### 3.3 Oral Microbiota and Immune-metabolic Interaction

Poor oral hygiene, often caused by improper cleaning techniques, promotes bacterial plaque accumulation. This buildup triggers gingivitis, which can progress to periodontitis if left untreated [1, 31]. This condition is driven by dysbiosis, or microbial imbalance, where harmful oral bacteria outgrow beneficial ones, thereby aggravating the disease. The relationship between oral microbiota, type 2 diabetes, and periodontitis is complex and interrelated [32]. The human body and its microbiome have co-evolved over millions of years, resulting in a symbiotic relationship that is crucial for maintaining health [33]. In the oral cavity, microbial communities form biofilms in various locations, including on teeth and dental restorations.

The formation of dental plaque is a structured process beginning with the development of a thin film known as the acquired pellicle. Initial bacterial adhesion occurs through weak physicochemical interactions with the pellicle, which is subsequently strengthened by specific adhesin–receptor binding. Secondary colonisers then attach to the primary bacteria, followed by bacterial proliferation and biofilm formation. Finally, bacterial detachment may occur. Maintaining a balance between the human microbiome and its host is crucial for overall health. Disruption of this balance due to external factors such as dietary habits, tobacco or alcohol use, stress, hormonal changes, puberty, poor oral hygiene, and diabetes can shift microbial communities from a symbiotic to a dysbiotic state, leading to diseases such as periodontitis [34].

Research indicates that gut and oral microbiota may contribute to the bidirectional relationship between diabetes and periodontal disease [35]. Type 2 diabetes is linked to changes in the oral microbiota, typically resulting in an increase in pathogenic species and a reduction in beneficial bacteria [31, 36-38]. For instance, studies have found that certain pathogenic bacteria, such as *Porphyromonas gingivalis* (*P. gingivalis*), are more prevalent in diabetic individuals, particularly those with periodontitis. This bacterium has been associated with poor periodontal outcomes, including increased clinical attachment loss and bleeding on probing, particularly in patients with uncontrolled diabetes [39].

**Fig. (1):**
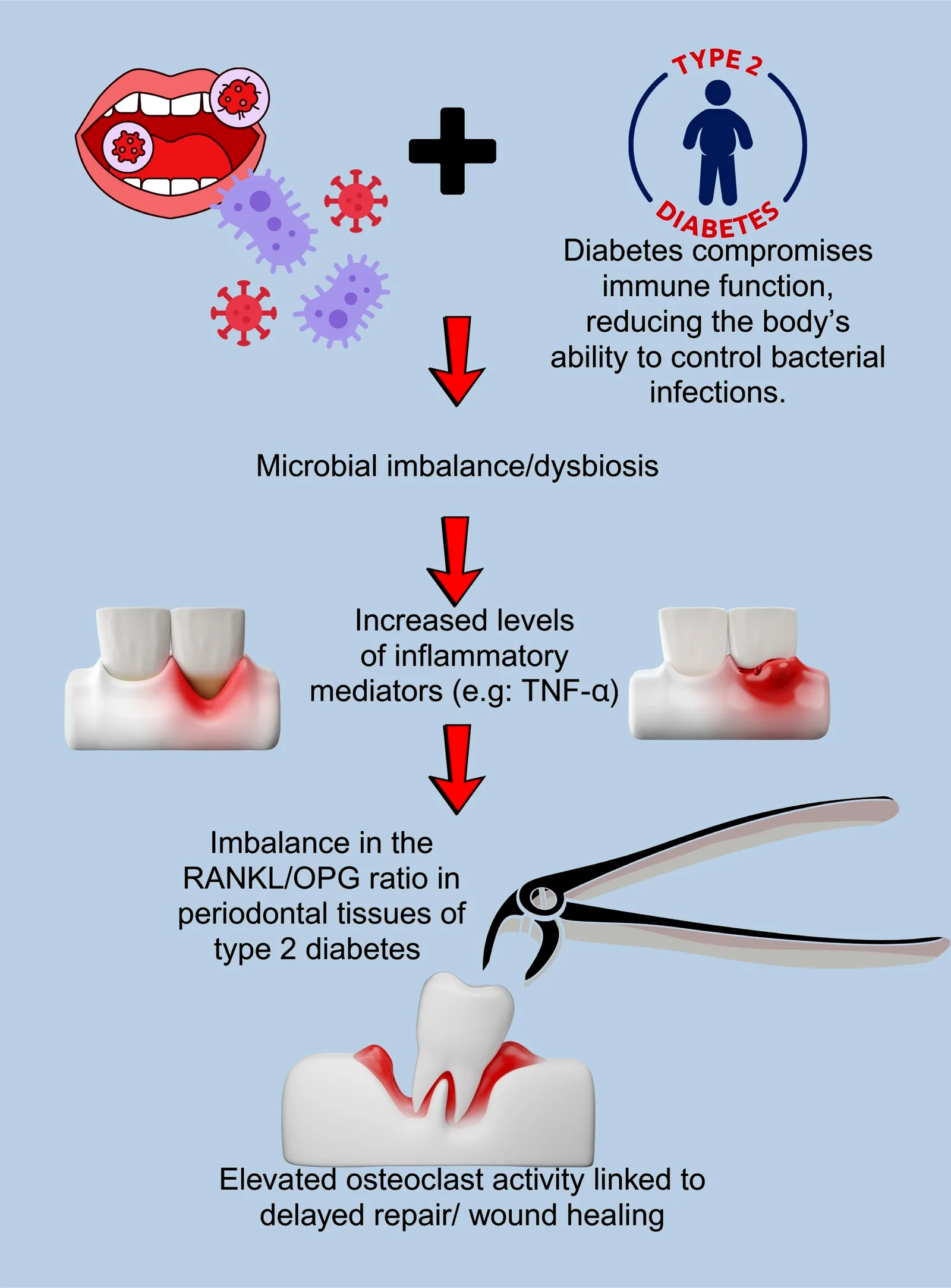
Association between type 2 diabetes and periodontal disease involving oral microbiota, inflammatory factors, and bone metabolism markers. Drawn with Canva software.

*P. gingivalis* can alter amino acid and lipid metabolism in periodontal tissues, producing metabolites such as short- chain fatty acids (SCFAs) [40] that modulate host immune responses. SCFA concentrations have been shown to be higher in periodontitis patients with type 2 diabetes [41]. These fatty acids can influence macrophage polarisation and T-cell differentiation, thereby modulating host immune responses [42]. In type 2 diabetes, pro-inflammatory cytokines reduce insulin receptor function on immune cells, causing insulin resistance and driving macrophages toward an M1 pro-inflammatory state [43]. Insulin resistance, the primary cause of type 2 diabetes, leads to hyperglycaemia, which elevates glucose levels in gingival crevicular fluid (GCF) [44]. This promotes harmful bacterial growth and biofilm formation while impairing immune responses, further worsening dysbiosis (Fig. **1**).

Furthermore, an increased prevalence of cariogenic and periodontopathogenic bacteria, including *Tannerella forsythia*, *Eubacterium nodatum*, *Parvimonas micra*, *Fusobacterium nucleatum* ssp., and *Prevotella intermedia*, has been observed in patients with type 2 diabetes [45]. A recent study by Carelli *et al.* (2023) identified the presence of both *Streptococcus mutans* and *Veillonella* spp. in individuals with poorer glycaemic control [46]. This highlights the interconnectedness of oral and gut microbiomes in the context of systemic health. Several studies suggest that some individuals may be more susceptible to dysbiosis due to affected immune system pathways, further complicating the relationship between diabetes, periodontitis, and the oral microbiota [47].

**Fig. (2):**
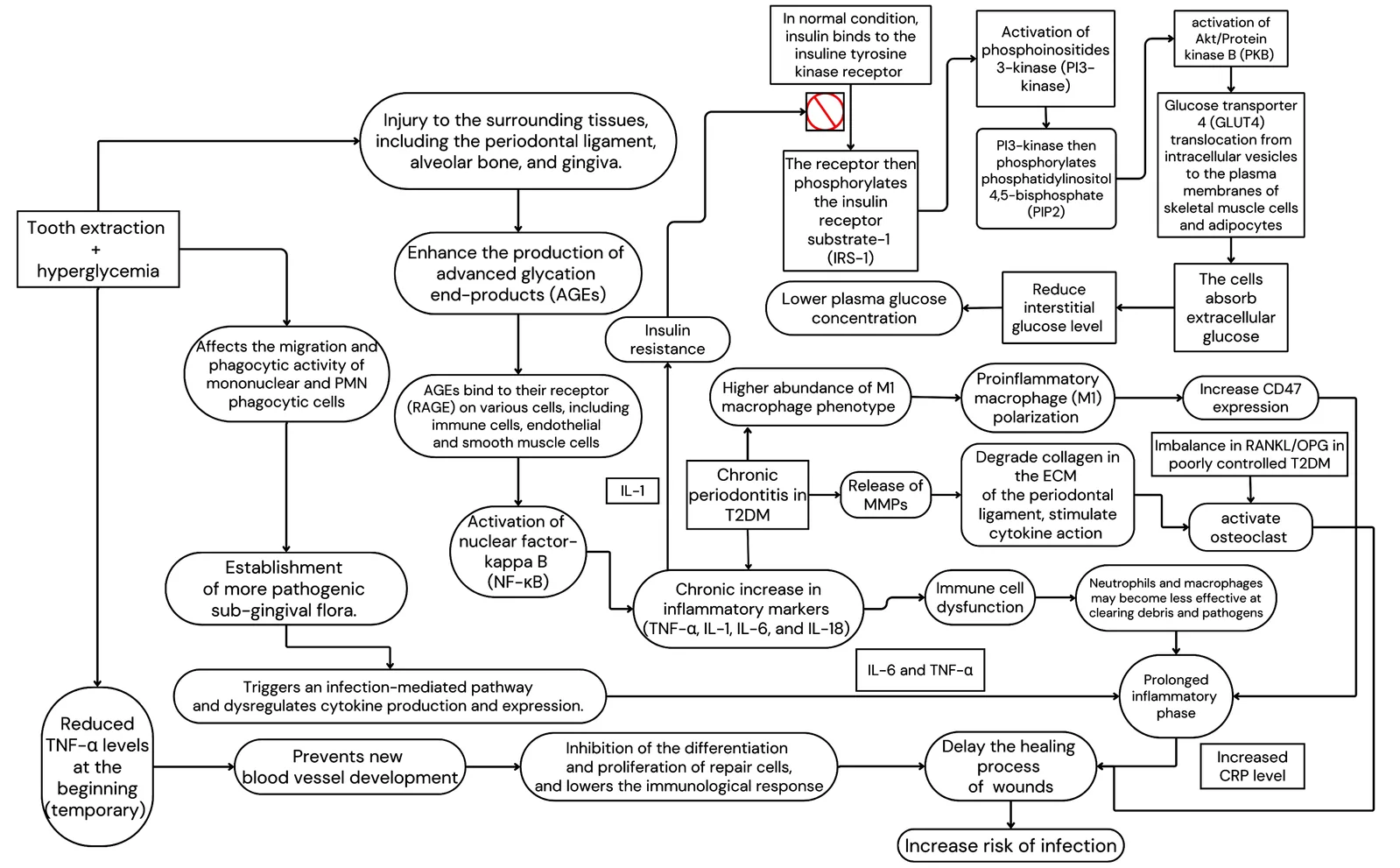
Pathway linking periodontal disease, insulin resistance in type 2 diabetes, inflammation, hyperglycaemia, and tooth extraction. Drawn with Canva software.

Recent immunophenotyping studies of gingival tissue demonstrate that samples from individuals with type 2 diabetes and periodontitis exhibit distinct immune cell profiles, including higher proportions of CD11b+ or CD4+ cells secreting IFNγ, IL-10, or IL-8, respectively, compared to periodontitis-alone samples [48]. Moreover, another study showed that patients with type 2 diabetes had a predominance of pro-inflammatory M1 macrophages at periodontally healthy sites compared to healthy sites in nondiabetic patients [49]. The regulation of chronic inflammation by macrophages, particularly through M1 polarisation, plays a crucial role in the development of diabetic complications. This may contribute to the increased susceptibility of individuals with diabetes to periodontitis (Fig. **2**) [49-51].

These functional shifts may be driven, in part, by microbiota-derived metabolites that influence immune cell metabolism, leading to sustained inflammatory signalling. This suggests that targeting microbial metabolites or their downstream immune effects may offer a potential therapeutic approach for managing periodontitis in diabetic patients. Similarly, maintaining good glycaemic control in diabetic patients can help prevent or slow the progression of periodontitis.

### 3.4 Tooth Extraction

Periodontal disease causes tissue destruction and loss of the alveolar bone and connective tissues supporting the teeth [52]. In cases where the supporting structures are severely damaged and the tooth becomes loose, extraction may be necessary to maintain oral health and prevent further complications.

In patients with diabetes, chronic hyperglycaemia leads to persistently elevated inflammatory markers. This sustained inflammatory state can paradoxically delay the healing of tooth extraction wounds by preventing progression from the inflammatory phase to subsequent stages of healing. High levels of inflammatory markers can impair immune cell function; for example, neutrophils and macrophages may become less effective at clearing debris and pathogens, prolonging the inflammatory phase. Diabetic oral wound healing is significantly affected by both increased inflammation and decreased production of new bone and connective tissue [53, 54].

Following tooth extraction, the body undergoes endocrine, immunological, and haematological changes, resulting in inflammation, proliferation, and tissue remodelling. Extraction also causes mechanical injury to surrounding tissues, including the gingiva, periodontal ligament, and alveolar bone. During healing, numerous substances are released that trigger a local immune response [55]. In patients with hyperglycaemia, delayed wound healing and increased susceptibility to infection after tooth extraction are commonly observed. These outcomes are influenced by underlying biological mechanisms associated with inflammation, bone metabolism, and oxidative stress markers.

#### 3.4.1 Inflammatory Markers

For patients with diabetes, particularly those with hyperglycaemia, the biological response to tooth extraction can be more complex and challenging due to the interplay between inflammation, impaired healing, and increased susceptibility to infections. Hyperglycaemia enhances the production of advanced glycation end products (AGEs), which bind to their receptors on various cells, including immune cells, endothelial cells, and smooth muscle cells [56, 57]. The binding of AGEs to RAGE triggers a cascade of pro-inflammatory signalling pathways, including activation of nuclear factor-kappa B (NF-κB) [58]. NF-κB is a transcription factor that upregulates the expression of several pro-inflammatory cytokines, such as TNF-α and IL-1 [59, 60] (Fig. **2**). Other cytokines that are chronically elevated in diabetic patients with periodontal disease include IL-6 and IL-18 [55, 61, 62].

##### 3.4.1.1 TNF-α

Proinflammatory cytokines, such as TNF-α, enhance wound healing by promoting angiogenesis, cellular proliferation, differentiation, growth, and immune responses [63]. Following tooth extraction, TNF-α is rapidly released by macrophages and other immune cells at the extraction site. Additionally, TNF-α plays a crucial role in necrosis and apoptosis, facilitating the clearance of damaged cells and foreign substances from wound sites [64]. Delayed healing of tooth extraction sockets is frequently reported in patients with uncontrolled diabetes [54, 65, 66].

A recent study investigating the levels of proinflammatory cytokines in the saliva of patients following tooth extraction found that TNF-α levels in non-diabetic patients (control group) increased two hours post-extraction and returned to baseline after two days. In contrast, patients with type 2 diabetes exhibited a delayed response, with TNF-α levels decreasing two hours post-extraction and then increasing two days after the procedure [63]. These findings suggest that patients with type 2 diabetes may exhibit delayed wound healing responses, characterised by minimal early inflammation but excessive, prolonged inflammation in chronic nonhealing wounds. This impaired inflammatory response can hinder angiogenesis, limit the differentiation and proliferation of repair cells, and suppress immune responses [63], thereby increasing the risk of post-extraction complications such as infections.

Chronic elevated TNF-α levels in patients with diabetes contribute to a state of persistent inflammation compared to non-diabetic individuals [55]. This continuous inflammatory response can prevent normal progression of the wound healing process from the inflammatory phase to the proliferative and remodelling phases.

##### 3.4.1.2 IL-1β

Interleukin-1β (IL-1β) is a key pro-inflammatory cytokine that plays a vital role in the body’s defence against infection and injury, as well as in facilitating the wound healing process. IL-1β is synthesised as an inactive precursor, pro-IL-1β, which accumulates in the cytosol and requires further processing to become active. The conversion of pro-IL-1β to its active form is primarily mediated by caspase-1, an enzyme activated within a multiprotein complex known as the inflammasome [67]. The activity of IL-1β must be carefully regulated, as excessive or prolonged expression can exacerbate inflammation and contribute to the pathogenesis of various inflammatory and autoimmune diseases [68].

A previous study using a Luminex multiplex assay found that salivary IL-1β concentrations in the type 2 diabetes group were significantly lower than those in the nondiabetic group, both at baseline and two hours after tooth extraction [63]. These findings suggest that type 2 diabetes influences the local expression of molecules that regulate anti-inflammatory responses and tissue healing. In contrast, Al Shehhi *et al.* (2024) reported higher IL-1β concentrations in human diabetic foot ulcers, which tend to decrease as the ulcers heal [69]. Moreover, elevated IL-1β levels have been associated with the development of insulin resistance and impaired wound healing in diabetes [70]. These observations underscore the importance of mechanisms that regulate IL-1β production and activity in maintaining homeostasis and promoting effective wound healing.

##### 3.4.1.3 IL-6

Interleukin-6 (IL-6) regulates inflammation and repair mechanisms. It is essential for the activation, differentiation, and function of endothelial cells, leukocytes, fibroblasts, and keratinocytes [71]. Inflammatory stimuli trigger macrophages to release IL-6, which recruits apoptotic leukocytes and activates T cells [72]. IL-6 also facilitates the replication, differentiation, and immunoglobulin production of pancreatic β-cells. As a mediator of the acute-phase response, IL-6 influences the production of proteins such as CRP, fibrinogen, haptoglobin, amyloid α, α-1-antitrypsin, and activates complement components [73]. Additionally, IL-6 exhibits immunomodulatory properties, initiating hyperinflammation mediated by polymorphonuclear leukocytes (PMNs), while paradoxically suppressing certain host immune responses.

IL-6 has the unique capacity to function as both a pro-inflammatory and an anti-inflammatory agent [74]. It can stimulate the production of anti-inflammatory cytokines such as IL-1 receptor antagonist (IL-1Ra) and IL-10 [75]. IL-1Ra blocks the activity of IL-1β, a potent pro-inflammatory cytokine, thereby mitigating inflammatory responses. IL-10 suppresses pro-inflammatory cytokine expression, inhibits antigen presentation, and reduces T-cell and macrophage activity. Through promoting the secretion of these cytokines, IL-6 helps dampen the inflammatory response. In certain contexts, IL-6 can also inhibit TNF-α production indirectly *via* enhanced IL-10 release, which suppresses TNF-α secretion [75].

Diabetes significantly alters the inflammatory landscape, impacting IL-6 behaviour during wound healing. In diabetic patients, the inflammatory response, including IL-6 activity, may be excessive or prolonged, resulting in delayed healing, poor wound closure, and increased risk of postoperative infections and complications. Following tooth extraction, diabetic patients exhibit a prolonged inflammatory response, with IL-6 levels continuing to rise two days post-procedure, whereas non-diabetic individuals show a transient IL-6 increase that declines after two days, reflecting quicker resolution of inflammation [63]. This prolonged inflammation in diabetic patients may contribute to delayed wound healing (Fig. **2**) and underscores the need for careful postoperative management to ensure optimal recovery and prevent complications.

##### 3.4.1.4 IL-18

Interleukin-18 (IL-18) is produced by dental pulp stem cells and functions as a pro-inflammatory cytokine, highlighting its role in immune responses within the dental pulp, particularly under inflammatory conditions. Dental pulp stem cells are integral to the immune defence and repair mechanisms of dental tissues [76]. By producing IL-18, these cells contribute to the inflammatory environment, as IL-18 stimulates interferon-gamma (IFN-γ) activity and activates both innate and adaptive immune responses [76]. IL-18 also promotes the overproduction of immune factors by activating macrophages, which can potentially disrupt normal immune system functions. Following tooth extraction, levels of IL-18 were higher in diabetic patients compared to healthy individuals, indicating an increased inflammatory burden in patients with diabetes [55].

#### 3.4.2 Oxidative Stress Markers

Oxidative stress and inflammation are closely interconnected. Inflammation can result from oxidative stress and simultaneously trigger the production of inflammatory mediators. These mediators, in turn, are activated by oxidative stress, creating an imbalance between oxidative stress and antioxidant levels [77]. Patients with diabetes mellitus exhibit reduced intracellular bacterial clearance and increased production of reactive oxygen species (ROS) [78]. ROS can promote the formation of advanced glycation end products (AGEs) and their receptors [53]. *In vitro* experiments have shown that elevated AGE levels increase extracellular MMP inducers and stimulate MMP secretion [79]. MMPs are detected in extracellular fluid and the extracellular matrix, and their activity leads to collagen breakdown, reducing bone strength. Furthermore, high AGE levels impair endothelial cell function and the extracellular matrix of the microvascular wall by covalently binding to active amino groups, which enhances oxidative stress and increases VEGF production [80]. Consequently, increased vascular permeability compromises blood flow and delays the healing process [53].

Total oxidant status (TOS) can be measured in serum or plasma. One study reported that TOS was significantly higher in diabetic patients than in healthy controls but decreased three months after tooth extraction. Total antioxidant capacity (TAC), measured in serum, plasma, or urine, was markedly reduced in diabetic patients and increased after the removal of inflammatory sources and oxidative stress generators in the oral cavity [55]. The post-extraction decrease in TOS suggests that removing the source of inflammation and infection can reduce oxidative stress levels. Another study found that diabetic patients had lower TAC and salivary catalase, whereas malondialdehyde (MDA) and TOS were significantly higher compared to healthy controls [81]. Post-extraction increases in TAC in both groups indicate an adaptive response to reduced oxidative stress. This improvement in antioxidant capacity may result from the removal of inflammatory foci, which reduces oxidative burden and allows endogenous antioxidant mechanisms to recover. Managing oxidative stress through interventions such as tooth extraction in diabetic patients can enhance antioxidant capacity and potentially mitigate some adverse effects associated with diabetes-related oxidative stress.

It is important to note that pre-analytical variables, such as sample collection methods, centrifugation conditions, and storage temperature, can influence measurements of oxidative stress markers like TOS and TAC. In our review, not all studies provided complete methodological details. While the lack of standardisation or incomplete reporting may contribute to variability in reported values, this limitation does not affect internal comparisons within individual studies (*e.g.*, between patient and control groups measured using the same protocols). However, it should be considered when interpreting findings across different studies.

#### 3.4.3 Bone Metabolism Markers

**Fig. (3):**
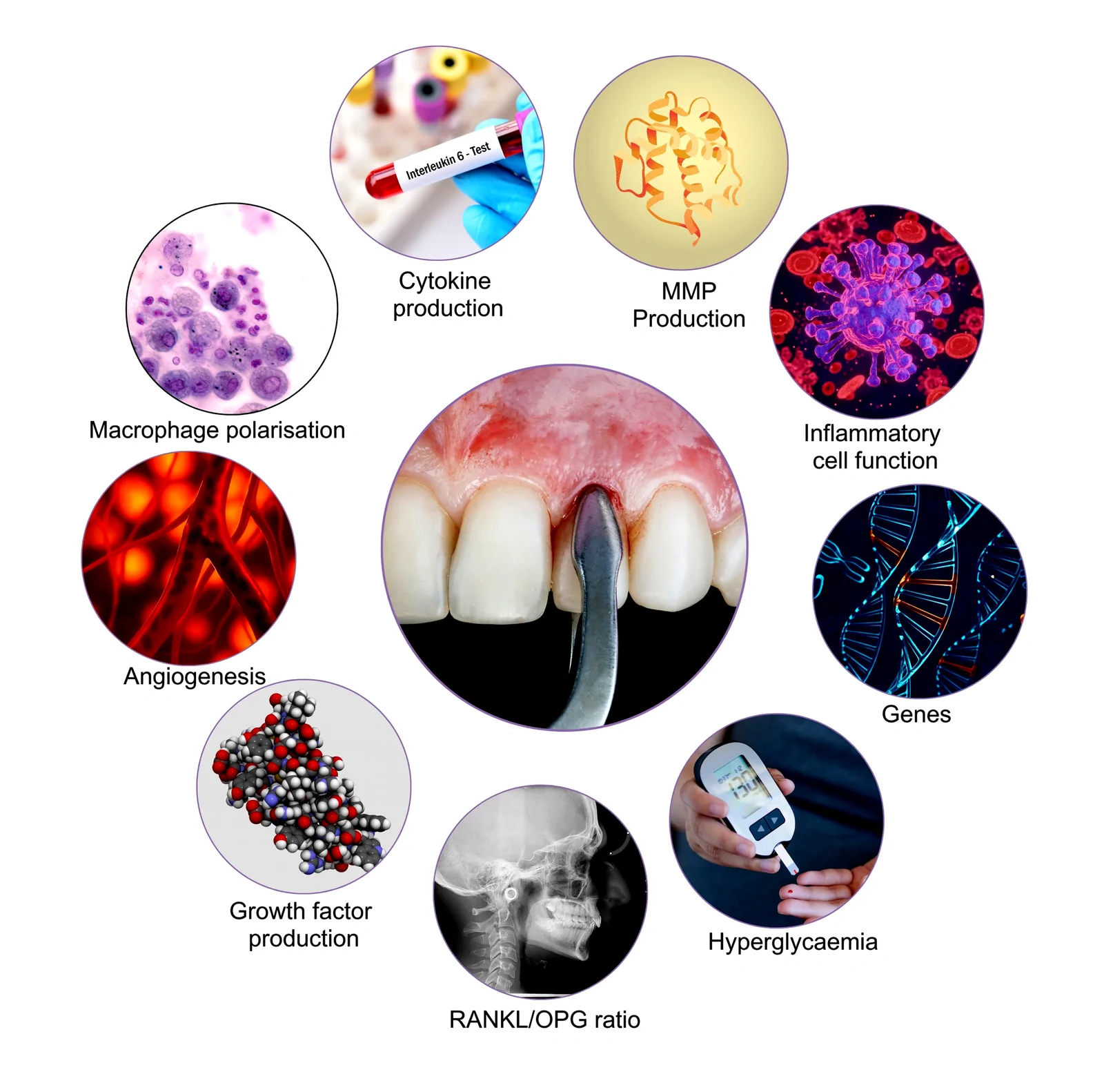
Factors involved in the bidirectional relationship between periodontal disease and type 2 diabetes.

Bone metabolism markers play a crucial role in understanding the mechanisms of socket wound healing after tooth extraction, particularly in patients with diabetes mellitus [53]. Delayed wound healing can result from abnormal cellular activity and dysregulation of growth factors and cytokines required to coordinate the normal healing process [53]. Bone formation and remodelling involve the proliferation and differentiation of osteocytes, along with the synthesis and mineralisation of the extracellular matrix [82]. These intricate processes are regulated by several cytokines, including insulin-like growth factor (IGF), transforming growth factor-β (TGF-β), vascular endothelial growth factor (VEGF), and bone morphogenetic protein (BMP) [82] (Fig. **3**).

The balance between components of the RANK/RANKL/OPG system is also critical for bone metabolism [83, 84]. Receptor activator of nuclear factor kappa B ligand (RANKL), a member of the tumour necrosis factor family, binds to the RANK receptor and is expressed in chondrocytes within the growth plates of bone. RANKL primarily stimulates osteoclast activity, and its binding to RANK can also activate dendritic and T cells, promoting bone resorption [85-89]. Osteoprotegerin (OPG), a soluble circulating molecule synthesised by osteoblasts and stromal cells, inhibits osteoclast differentiation by binding to RANKL [85, 90, 91]. High OPG levels may further promote the differentiation of pre-osteoblasts into mature osteoblasts [92]. Normal bone metabolic activity and the maintenance of bone mass depend on a balance between RANKL and OPG [93].

In patients with poorly controlled type 2 diabetes, a persistent imbalance in the RANKL/OPG ratio—characterised by elevated RANKL and reduced OPG—enhances osteoclast differentiation and activity, accelerating alveolar bone resorption and delaying wound healing [94] (Fig. **2**). Conversely, higher OPG levels relative to RANKL inhibit osteoclast activation, providing protection against bone loss. In the referenced study, these markers were measured in gingival crevicular fluid (GCF), which reflects local periodontal tissue conditions more directly than systemic samples such as serum. This distinction is important, as biomarker concentrations and RANKL/OPG ratios may differ between local and systemic sources due to site-specific pathophysiological processes.

### 3.5 Role of Genes

Genes play a significant role in the predisposition to and progression of periodontitis and diabetes mellitus. The contribution of genetic predisposition to the development of periodontitis among diabetic patients is becoming increasingly evident, with the identification of specific gene variants providing deeper insights into the complex relationship between these conditions.

Genetic research has identified specific genes linking periodontitis with diabetes, offering new insights into their comorbidity. One study investigated the association between Peroxisome Proliferator-Activated Receptor Gamma (PPARG) gene, single nucleotide polymorphisms (SNPs) and periodontitis and type 2 diabetes in the Brazilian population [95]. The rs1801282 SNP was not associated with either condition, whereas the G allele of rs1151999 in the PPARG gene was significantly associated with a reduced risk of developing periodontitis alongside diabetes, with CCGT haplotype carriers being twice as likely to develop the condition.

Further studies have identified several key genes influencing both periodontitis and diabetes. One study reported 79 common differentially expressed genes associated with both conditions [96]. Using a multi-omics integration approach, six candidate genes were highlighted, with Ras-Associated Protein 2A (RAP2A) and Mitochondrial Calcium Uniporter Regulator 1 (MCUR1) identified as high-risk genes, and WNK1, NFIX, FOS, and PANX1 as protective genes.

Gene-environment interactions also influence disease risk. A study in the Chinese population examined the impact of adiponectin (ADIPOQ-rs1501299) and leptin receptor (LEPR-rs1137100) polymorphisms on the risk of type 2 diabetes associated with periodontitis [97]. Among 239 subjects, significant associations were found between moderate/severe periodontitis and these variants after adjustment for covariates.

European studies further support the genetic link between periodontitis and diabetes. Positive global genetic correlations were observed between periodontitis and type 2 diabetes or glycaemic traits, with pleiotropic SNPs identified through pairwise colocalization analysis. Bidirectional Mendelian randomisation (MR) analysis revealed that periodontitis was significantly associated with fasting glucose (FG), Homeostasis Model Assessment of Beta Cell Function (HOMA-β), and HOMA-β adjusted for BMI (HOMA-βadjBMI), suggesting shared biological pathways [98].

Cytokine-related gene polymorphisms also play a role. The IL-1B -511 TT genotype was more frequent in patients with diabetes, while the CC genotype was strongly associated with periodontitis. IL-1A -889 TT and IL-1B +3954 TT genotypes increased the risk of periodontitis in both diabetic and non-diabetic patients [99]. Similarly, polymorphisms in RBMS1 (rs7593730), AGER (rs184003), and VEGFA (rs9472138) have been linked to periodontitis and type 2 diabetes, with RBMS1 rs7593730 TT genotype associated with increased risk of periodontitis but a reduced risk for comorbid periodontitis and diabetes [100].

Hub genes linking periodontitis and diabetes, such as Human Beta-Defensin (HBD) and Absent in Melanoma 2 (AIM2), may serve as diagnostic markers. The NOD-like receptor signalling pathway was identified as a central mechanism connecting the two diseases, with hub genes NLRC4, AIM2, NLRP2, and HBD implicated in the periodontal disease–type 1 diabetes pair, and IL-1B, AIM2, NLRP2, and HBD in the periodontal disease–type 2 diabetes pair. Gene set variation analysis (GSVA) revealed more active Reactome signalling pathways in periodontitis compared to diabetes, and distinct immunocyte infiltration patterns were observed. Network analysis showed that AIM2 and HBD target genes were involved in the NOD-like receptor signalling pathway, demonstrating their potential diagnostic value [101].

Additional hub genes identified include Protein Tyrosine Phosphatase Receptor Type C (PTPRC), Hepatocyte Growth Factor (HGF), Rac Family Small GTPase 2 (RAC2), and Inositol Polyphosphate-5-Phosphatase D (INPP5D), which may serve as therapeutic targets [102]. Furthermore, four ferroptosis-related core genes—IL-1β, IL-6, Nuclear Factor Erythroid 2-Like 2 (NFE2L2), and Arachidonate 5-Lipoxygenase (ALOX5)—have been proposed as potential biomarkers for the clinical diagnosis and management of periodontitis in patients with type 2 diabetes [103].

In addition to genetic factors, epigenetic modifications have also been implicated in the association between type 2 diabetes and periodontal disease. An animal study investigated epigenetic changes in gingival tissue in streptozotocin-induced diabetic pigs [104]. The study found that DNA methylation alterations in the gingival tissue of diabetic animals were associated with various biological processes, cellular components, molecular functions, and signalling pathways related to morphological changes and the status of periodontal tissues.

## 4 FUTURE DIRECTION

The intricate relationship between periodontal disease and type 2 diabetes has been a subject of considerable research, revealing a bidirectional association in which each condition exacerbates the other. As our understanding of these diseases deepens, future research must delve into the molecular mechanisms and pathways that underpin this association. The future directions in research on the molecular mechanisms and pathways related to the association between periodontal disease and type 2 diabetes involve several promising areas, including advanced molecular techniques, novel therapeutic targets, personalised medicine, and improved understanding of the microbiome.

In advanced molecular techniques, current research focuses on genomic and epigenomic studies. Next-generation sequencing and epigenomic profiling are among the high-demand laboratory research areas aimed at identifying genetic and epigenetic factors that contribute to both conditions [105]. Other research areas include transcriptomics and proteomics, which use RNA sequencing and proteomic analyses to identify differentially expressed genes and proteins that mediate the interaction between periodontal disease and diabetes [106].

Novel therapeutic targets explore new molecules, pathways, or mechanisms within the body that can serve as points of intervention for developing new treatments. These targets can include proteins, genes, or other biomolecules that play a crucial role in disease progression. The association between periodontitis and type 2 diabetes is well-documented, with each condition potentially exacerbating the other.

Developing targeted anti-inflammatory therapies that specifically modulate the cytokines and pathways involved in both conditions—for example, targeting TNF-α, IL-1β, and IL-6, which are expected to be elevated in both periodontitis and type 2 diabetes—can help reduce systemic inflammation and improve both conditions [107, 108]. Advanced glycation end-products are elevated in diabetes and contribute to chronic inflammation and tissue damage [109]. Therefore, targeting the AGE-RAGE axis can help mitigate periodontal inflammation and improve glycaemic control. Another promising approach involves exploring the use of antioxidants, compounds that reduce oxidative stress, such as N-acetylcysteine (NAC) or vitamin E, which can help alleviate the oxidative damage seen in both periodontitis and type 2 diabetes.

The role of the microbiome in the pathogenesis of periodontal disease and type 2 diabetes presents another promising research direction [36, 110]. Alterations in the oral and gut microbiomes have been implicated in both conditions, suggesting a potential link between microbial dysbiosis and disease progression. Future research should explore how changes in the oral and gut microbiomes contribute to the bidirectional relationship between periodontal disease and type 2 diabetes [111]. Understanding microbiome-host interactions and their impact on immune responses could lead to innovative therapeutic strategies, including the use of probiotics and prebiotics to modulate the microbiome.

Personalised medicine plays a significant role in managing the association between periodontitis and type 2 diabetes by tailoring prevention, diagnosis, and treatment strategies to the individual characteristics of each patient. By identifying and validating biomarkers that can predict the risk and progression of periodontal disease and diabetes, these biomarkers can be used for early diagnosis, monitoring treatment response, and tailoring personalised therapeutic strategies. Current approaches in genetic profiling can identify the risk for both periodontitis and type 2 diabetes due to genetic predispositions [112]. For example, certain genetic variants may influence inflammatory responses, insulin resistance, or susceptibility to specific oral pathogens. Understanding genetic abnormalities can also guide the selection of specific treatments. For instance, individuals with certain genetic defects may respond better to anti-inflammatory drugs or require more aggressive periodontal treatment.

Another area being explored is immune system modulation. Immune modulators are being developed to enhance the body's ability to control periodontal infections and reduce systemic inflammation. In addition, there is potential to explore vaccines against key periodontal pathogens or inflammatory mediators involved in both periodontal disease and diabetes.

Apart from this, personalised dietary plans based on individual metabolic profiles and preferences can help manage both type 2 diabetes and periodontitis. For example, diets rich in anti-inflammatory foods and low in processed sugars can benefit both conditions [113]. Personalised recommendations for oral hygiene routines, including specific toothpastes, mouthwashes, and dental care practices, can be made based on individual periodontal health status and risks.

Research in these areas is ongoing, and while some therapies are already in use or in clinical trials, others are still in the experimental stages. Future research directions aim to deepen our understanding of the molecular interplay between periodontal disease and type 2 diabetes. By leveraging advanced molecular techniques, identifying novel therapeutic targets, and adopting personalised medicine approaches, researchers hope to develop more effective strategies for preventing and managing these interconnected conditions. Interdisciplinary collaboration and consideration of lifestyle and environmental factors will also be crucial in addressing the complex interactions between these diseases.

## CONCLUSION

Periodontal disease causes inflammation and infection of the gums, leading to progressive destruction of soft tissue and alveolar bone, which may ultimately result in tooth loss if left untreated. Type 2 diabetes exacerbates this condition through oxidative stress, increased cytokine levels, fibroblast apoptosis, and impaired angiogenesis, all of which hinder gingival healing. Hyperglycaemia in type 2 diabetes also promotes bacterial growth, while persistent TNF-α levels drive chronic inflammation and tissue destruction. TNF-α plays a crucial role in necrosis and apoptosis; thus, the use of TNF inhibitors may reduce inflammation and support new bone and osteoid formation. In addition, the RANKL/OPG pathway, which governs osteoclastogenesis and bone resorption, is tightly regulated by inflammatory mediators. A persistent imbalance in the RANKL/OPG ratio in the periodontal tissues of patients with type 2 diabetes results in increased osteoclastic activity, which negatively affects wound healing. Hence, future drug designs could be developed to target these pathways. This review elucidates the molecular mechanisms of the oral microbiota–inflammatory factors–bone metabolism marker axis in the bidirectional link between periodontal disease and type 2 diabetes. Investigations focusing on bone resorption and inflammatory biomarkers may therefore improve the diagnosis and management of periodontal disease in patients with type 2 diabetes.

## STUDY LIMITATIONS

While this review provides insight into the molecular mechanisms and pathways related to the association between periodontal disease and type 2 diabetes, certain inherent limitations should be acknowledged. This review was designed to provide a broad and integrative overview of existing evidence rather than a systematic or quantitative synthesis. The selection of studies was guided by relevance and thematic focus, which may not encompass all available literature on the topic.

It is important to note that the biomarkers included in this review are among those commonly reported in the literature and are related to oxidative stress, inflammation, bone metabolism, and genes. Therefore, the review may not cover all biomarkers that might be relevant, especially if they have not been widely studied or highlighted in the literature. Ongoing exploration is needed to identify additional molecular players that may contribute to the processes discussed in this paper due to the evolving nature of the field. In the future, potential bias can be minimised by adopting more structured and methodical approaches to literature evaluation. A broader inclusion of biological processes and the latest research will contribute to a more comprehensive understanding of the topic.

## AUTHOR’S CONTRIBUTIONS

The authors confirm their contribution to this paper as follow: S.R.M.Z. contributed to the conceptualisation, literature review, and writing of the manuscript. H.A.I., W.M.W.M., N.M., N.K.K., T.N.N.T.I., W.S.W.A.R., and Z.M.Y. contributed to the literature review and writing of the manuscript. S.D. contributed to the revision of the manuscript. All authors have read and improved the final version of the manuscript.

## Figures and Tables

**Table 1:** Interrelationship between periodontal disease and type 2 diabetes.

Serial Number	Study Design	Subject/Patient	Key Findings	References
1	Observational study	Human sample	Higher GCF AGEs in periodontitis with type 2 diabetes compared to healthy controls.	[27]
2	Epidemiological study	Human data	Smoking impairs periodontal health in Korean adults, but no added effect with diabetes.	[30]
3	Observational study	Human sample	Type 2 diabetes and periodontitis are linked; poor oral hygiene associated with *P. gingivalis* and poor glycaemic control.	[31]
4	Observational study	Human sample	Pathogenic species are abundant in both healthy and periodontitis states of type 2 diabetes, indicating a higher risk of disease progression.	[38]
5	Observational study	Human sample	Higher salivary *P. gingivalis* in uncontrolled DM; linked to worse periodontal outcomes.	[39]
6	*In vivo* study	Mice	Periodontal disease shows impaired glucose metabolism and disrupts microbial balance.	[35]
7	Observational study	Human sample	Higher SCFAs in type 2 diabetes with periodontitis indicate increased disease risk.	[41]
8	Observational study	Human sample	Poor glycaemic control is associated with increased levels and frequencies of periodontal pathogens in the subgingival biofilm of subjects with type 2 diabetes and generalised chronic periodontitis.	[45]
9	Observational study	Human sample	Type 2 diabetes with periodontitis has distinct immune profiles, with more CD11b^+^ IFNγ/IL-10 and CD4^+^ IL-8 cells than periodontitis alone.	[48]
10	Observational study	Human sample	Type 2 diabetes patients show more pro-inflammatory M1 macrophages at healthy periodontal sites than non-diabetics.	[49]
11	*In vivo* study	Mice	Delayed healing of tooth extraction sockets in poorly controlled diabetes.	[54]
12	Observational study	Human sample	Salivary oxidative and proinflammatory biomarkers in diabetics correlate with HbA1c and may indicate post-extraction healing status.	[55]
13	Observational study	Human sample	Salivary IL-17A was strongly associated with periodontitis, whereas salivary IL-18 was associated with FPG, and serum IL-18 was associated with HbA_1C_.	[61]
14	Observational study	Human sample	The type 2 diabetes group showed a delayed TNF-α response, resulting in delayed wound healing after extraction.	[63]
15	Observational study	Human sample	In poorly controlled type 2 diabetes, an elevated RANKL/OPG ratio promotes osteoclast activity, speeding alveolar bone loss and delaying wound healing.	[94]
16	Retrospective cohort study	Human sample	Diabetic patients showed lower TAC and salivary catalase, but higher MDA and TOS than healthy controls.	[81]
17	Observational study	Human sample	rs1801282 showed no association, while the G allele of rs1151999 in PPARG reduced the risk of periodontitis with diabetes; CCGT haplotype carriers had double the risk.	[95]
18	Bioinformatic and Mendelian randomization study	Human dataset	RAP2A and MCUR1 have been identified as high-risk genes, while WNK1, NFIX, FOS, and PANX1 have been identified as protective genes in periodontitis-related type 2 diabetes.	[96]
19	Observational study	Human sample	A significant association between moderate/severe periodontitis and variants in rs1501299 and rs1137100 was found after adjustment of the covariates.	[97]
20	Bioinformatic study	Human study	Colocalization analysis identified shared SNPs between periodontitis and type 2 diabetes/glycaemic traits, and MR analysis linked periodontitis to fasting glucose and HOMA-β measures.	[98]
21	Observational study	Human sample	Only IL-1B -511 genotypes differed: TT was more frequent in diabetics, CC linked to periodontitis, and TT was protective but possibly neutralized in diabetes.	[99]
22	Observational study	Human sample	RBMS1 rs7593730 TT genotype increased periodontitis risk 2.29-fold but reduced risk of periodontitis with type 2 diabetes by ~70%.	[100]
23	Bioinformatic study	GEO (Gene Expression Omnibus) datasets	Hub genes NLRC4, AIM2, NLRP2, and HBD were linked to periodontitis–Type 1 diabetes, and IL-1B, AIM2, NLRP2, and HBD to periodontitis–Type 2 diabetes.	[101]
24	Observational study	Human sample	PTPRC, HGF, RAC2, and INPP5D were identified as hub genes linking periodontitis and type 2 diabetes, and potential therapeutic targets.	[102]
25	Bioinformatic study	Human dataset	Four ferroptosis-related genes (IL-1β, IL-6, NFE2L2, ALOX5) were identified as potential biomarkers for periodontitis with type 2 diabetes.	[103]
26	*In vivo* study	Pigs	DNA methylation changes in gingival tissue of diabetics were linked to cellular functions, pathways, and periodontal tissue morphology.	[104]

## LIST OF ABBREVIATIONS

AAP: American Academy of Periodontology
ADIPOQ: Adiponectin
AGER: Advanced Glycation End-product Receptor
AGEs: Advanced Glycation End-products
AIM2: Absent in Melanoma 2
PKB: Protein Kinase B
ALOX5: Arachidonate 5-Lipoxygenase
BBS7: Bardet-Biedl Syndrome 7
BMP: Bone Morphogenetic Protein
CD11b+: Cluster of Differentiation 11b Positive Cells
CD34: Cluster of Differentiation 34
CD4+: Cluster of Differentiation 4 Positive Cells
CRP: C-reactive Protein
CXC: C-X-C Motif Chemokine
DM: Diabetes Mellitus
DNA: Deoxyribonucleic Acid
EGR1: Early Growth Response 1
FG: Fasting Glucose
FMOD: Fibromodulin
GCF: Gingival Crevicular Fluid
GLUT4: Glucose Transporter Type 4
GSVA: Gene Set Variation Analysis
HbA1c: Glycated Haemoglobin
HBD: Human BetaDefensin
HGF: Hepatocyte Growth Factor
HOMAβ: Homeostasis Model Assessment of Beta Cell Function
HOMAβadjBMI: HOMA of Beta Cell Function adjusted for Body Mass Index
IDF: International Diabetes Federation
IFNγ: Interferongamma
IGF2: Insulinlike Growth Factor 2
IL18: Interleukin18
IL1A: Interleukin 1 Alpha
IL1β: Interleukin1 Beta
IL6: Interleukin6
INPP5D: Inositol Polyphosphate5Phosphatase D
IRS1: Insulin Receptor Substrate1
LEPR: Leptin Receptor
LPS: Lipopolysaccharide
MCUR1: Mitochondrial Calcium Uniporter Regulator 1
MDA: Malondialdehyde
MMPs: Matrix Metalloproteinases
MAF: Minor Allele Frequencies
mRNA: Messenger Ribonucleic Acid
NFκB: Nuclear Factor Kappa B cells
NHMS: National Health and Morbidity Survey
NLRC4: NODlike Receptors C4
NOD: Nucleotidebinding Oligomerization Domain
NFE2L2: Nuclear Factor Erythroid 2Like 2
OPG: Osteoprotegerin
OPGL: Osteoprotegerin Ligand
PI3kinase: Phosphoinositide 3kinase
PIP2: Phosphatidylinositol 4 5bisphosphate
PPARG: Peroxisome ProliferatorActivated Receptor Gamma
RAC2: Rac Family Small GTPase 2
RAGE: Receptor for Advanced Glycation Endproducts
RANK: Receptor Activator of Nuclear Factor κB
RANKL: Receptor Activator of Nuclear Factor κB Ligand
RAP2A: RasAssociated Protein 2A
RBMS1: RNA Binding Motif Protein 1
ROS: Reactive Oxygen Species
scRNAseq: SingleCell RNA Sequencing
SCFAs: Shortchain
SNPs: Single Nucleotide Polymorphisms
TOS: Total oxidative status
TAC: Total Antioxidant Capacity
T1D: Type 1 Diabetes
TAC: Total Antioxidant Capacity
TGFΒ: Transforming Growth Factor Beta
TNF: Tumour Necrosis Factor
TNFα: Tumour Necrosis Factor alpha
TOS: Total Oxidative Status
TRIzol: TriReagent
TXN: Thioredoxin
USD: United States Dollars
VEGFA: Vascular Endothelial Growth Factor A

## References

[1] Gasner N.S., Schure R.S. (2023). Periodontal disease..

[2] Nazir M.A. (2017). Prevalence of periodontal disease, its association with systemic diseases and prevention.. Int. J. Health Sci..

[3] Anuwar A.H.K., Ng C.W., Safii S.H., Saub R., Ab-Murat N. (2024). Modelling the national economic burden of non-surgical periodontal management in specialist clinics in Malaysia using a markov model.. BMC Oral Health.

[4] Rafat Kotb S.H. (2023). Overview of periodontal disease, etiology, pathogenesis, diagnosis and treatment therapy.. Open Access J. Dent. Sci..

[5] Banday M.Z., Sameer A.S., Nissar S. (2020). Pathophysiology of diabetes: An overview.. Avicenna J. Med..

[6] Sacks D.B., Arnold M., Bakris G.L., Bruns D.E., Horvath A.R., Lernmark Å., Metzger B.E., Nathan D.M., Kirkman M.S. (2023). Guidelines and recommendations for laboratory analysis in the diagnosis and management of diabetes mellitus.. Clin. Chem..

[7] ElSayed N.A., McCoy R.G., Aleppo G., Balapattabi K., Beverly E.A., Briggs Early K., Bruemmer D., Ebekozien O., Echouffo-Tcheugui J.B., Ekhlaspour L., Gaglia J.L., Garg R., Khunti K., Lal R., Lingvay I., Matfin G., Pandya N., Pekas E.J., Pilla S.J., Polsky S., Segal A.R., Seley J.J., Selvin E., Stanton R.C., Bannuru R.R. (2025). 2. Diagnosis and classification of diabetes: Standards of care in diabetes—2025.. Diabetes Care.

[8] (2014). Diagnosis and classification of diabetes mellitus.. Diabetes Care.

[9] ElSayed N.A., Aleppo G., Bannuru R.R., Bruemmer D., Collins B.S., Ekhlaspour L., Gaglia J.L., Hilliard M.E., Johnson E.L., Khunti K., Lingvay I., Matfin G., McCoy R.G., Perry M.L., Pilla S.J., Polsky S., Prahalad P., Pratley R.E., Segal A.R., Seley J.J., Selvin E., Stanton R.C., Gabbay R.A. (2024). 2. Diagnosis and classification of diabetes: Standards of care in diabetes—2024.. Diabetes Care.

[10] Akhtar S., Nasir J.A., Ali A., Asghar M., Majeed R., Sarwar A. (2022). Prevalence of type-2 diabetes and prediabetes in Malaysia: A systematic review and meta-analysis.. PLoS One.

[11] Standl E., Khunti K., Hansen T.B., Schnell O. (2019). The global epidemics of diabetes in the 21st century: Current situation and perspectives.. Eur. J. Prev. Cardiol..

[12] (2021). The IDF Diabetes Atlas.. https://diabetesatlas.org/.

[13] (2023). Non-communicable diseases and healthcare demand - Key findings.. https://iku.nih.gov.my/images/nhms2023/key-findings-nhms-2023.pdf.

[14] Ganapathy SS, Aris TH, Ahmad NA (2019). Non-communicable diseases: Diabetes, hypertension and hypercholesterolaemia.. https://iku.moh.gov.my/images/IKU/Document/REPORT/NHMS2019/Report_NHMS2019-NCD_v2.pdf.

[15] (2021). Diabetes in Malaysia 2021.. https://idf.org/our-network/regions-and-members/western-pacific/members/malaysia/.

[16] Kumari A., Sodum N., Ravichandiran V., Kumar N. (2023). Role of SIRT-1 as a target for treatment and prevention of diabetic nephropathy: A review.. Curr. Mol. Pharmacol..

[17] Aikaeli F., Njim T., Gissing S., Moyo F., Alam U., Mfinanga S.G., Okebe J., Ramaiya K., Webb E.L., Jaffar S., Garrib A. (2022). Prevalence of microvascular and macrovascular complications of diabetes in newly diagnosed type 2 diabetes in low-and-middle-income countries: A systematic review and meta-analysis.. PLOS Glob. Public Health.

[18] Genco R.J., Graziani F., Hasturk H. (2020). Effects of periodontal disease on glycemic control, complications, and incidence of diabetes mellitus.. Periodontol. 2000.

[19] Graves D.T., Ding Z., Yang Y. (2020). The impact of diabetes on periodontal diseases.. Periodontol. 2000.

[20] Roden M., Shulman G.I. (2019). The integrative biology of type 2 diabetes.. Nature.

[21] Galicia-Garcia U., Benito-Vicente A., Jebari S., Larrea-Sebal A., Siddiqi H., Uribe K.B., Ostolaza H., Martín C. (2020). Pathophysiology of type 2 diabetes mellitus.. Int. J. Mol. Sci..

[22] Bains V.K., Mahendra J., Mahendra L., Mittal M., Valli G. (2022). Markers, pathways, and current evidence for periodontitis-associated insulin resistance: A narrative review.. J. Int. Soc. Prev. Community Dent..

[23] Kato S., Tanabe N., Nagao M., Sekino J., Tomita K., Sakai M., Abe K., Suzuki N., Ueda K. (2020). Glucose transporter 4 mediates LPS-induced IL-6 production in osteoblasts under high glucose conditions.. J. Oral Sci..

[24] Dandona P., Aljada A., Bandyopadhyay A. (2004). Inflammation: The link between insulin resistance, obesity and diabetes.. Trends Immunol..

[25] Casanova L., Hughes F.J., Preshaw P.M. (2014). Diabetes and periodontal disease: A two-way relationship.. Br. Dent. J..

[26] Plemmenos G., Piperi C. (2022). Pathogenic molecular mechanisms in periodontitis and peri-implantitis: Role of advanced glycation end products.. Life.

[27] Akram Z., Alqahtani F., Alqahtani M., Al-Kheraif A.A., Javed F. (2020). Levels of advanced glycation end products in gingival crevicular fluid of chronic periodontitis patients with and without type-2 diabetes mellitus.. J. Periodontol..

[28] Silva H. (2021). Tobacco use and periodontal disease—The role of microvascular dysfunction.. Biology.

[29] Zhang W., Fang M., Song F., Windsor L.J. (2011). Effects of cigarette smoke condensate and nicotine on human gingival fibroblast-mediated collagen degradation.. J. Periodontol..

[30] Han D.H., Lim S., Kim J.B. (2012). The association of smoking and diabetes with periodontitis in a Korean population.. J. Periodontol..

[31] Radhakrishnan P., Anbalagan R., Barani R., Mani M., Seshadri K.G., Srikanth P. (2019). Sequencing of *Porphyromonas gingivalis* from saliva in patients with periodontitis and type 2 diabetes mellitus.. Indian J. Med. Microbiol..

[32] Radaic A., Kapila Y.L. (2021). The oralome and its dysbiosis: New insights into oral microbiome-host interactions.. Comput. Struct. Biotechnol. J..

[33] Samaranayake L., Matsubara V.H. (2017). Normal oral flora and the oral ecosystem.. Dent. Clin. North Am..

[34] Kilian M., Chapple I.L.C., Hannig M., Marsh P.D., Meuric V., Pedersen A.M.L., Tonetti M.S., Wade W.G., Zaura E. (2016). The oral microbiome – An update for oral healthcare professionals.. Br. Dent. J..

[35] Li L., Bao J., Chang Y., Wang M., Chen B., Yan F. (2021). Gut microbiota may mediate the influence of periodontitis on prediabetes.. J. Dent. Res..

[36] Qin H., Li G., Xu X., Zhang C., Zhong W., Xu S., Yin Y., Song J. (2022). The role of oral microbiome in periodontitis under diabetes mellitus.. J. Oral Microbiol..

[37] Păunică I., Giurgiu M., Dumitriu A.S., Păunică S., Pantea Stoian A.M., Martu M.A., Serafinceanu C. (2023). The bidirectional relationship between periodontal disease and diabetes mellitus—A review.. Diagnostics.

[38] Shi B., Lux R., Klokkevold P., Chang M., Barnard E., Haake S., Li H. (2020). The subgingival microbiome associated with periodontitis in type 2 diabetes mellitus.. ISME J..

[39] Aoyama N., Suzuki J., Kobayashi N., Hanatani T., Ashigaki N., Yoshida A., Shiheido Y., Sato H., Izumi Y., Isobe M. (2018). Increased oral *Porphyromonas gingivalis* prevalence in cardiovascular patients with uncontrolled diabetes mellitus.. Int. Heart J..

[40] Sun J., Wang X., Xiao J., Yang Q., Huang X., Yang Z., Liu H., Liu Y., Wang H., Huang Z., Ma L., Cao Z. (2024). Autophagy mediates the impact of *Porphyromonas gingivalis* on short-chain fatty acids metabolism in periodontitis-induced gut dysbiosis.. Sci. Rep..

[41] Lu X., Liu T., Zhou J., Liu J., Yuan Z., Guo L. (2022). Subgingival microbiome in periodontitis and type 2 diabetes mellitus: An exploratory study using metagenomic sequencing.. J. Periodontal Implant Sci..

[42] Ney L.M., Wipplinger M., Grossmann M., Engert N., Wegner V.D., Mosig A.S. (2023). Short chain fatty acids: Key regulators of the local and systemic immune response in inflammatory diseases and infections.. Open Biol..

[43] Berbudi A., Khairani S., Tjahjadi A. (2025). Interplay between insulin resistance and immune dysregulation in type 2 diabetes mellitus: Implications for therapeutic interventions.. ImmunoTargets Ther..

[44] Patel C., Dave B., Patel R., Kumar S., Dattani V., Joshi S., Haque M. (2023). Gingival crevicular blood glucose as a novel method for screening diabetes mellitus in periodontally compromised patients.. Cureus.

[45] Miranda T.S., Feres M., Retamal-Valdés B., Perez-Chaparro P.J., Maciel S.S., Duarte P.M. (2017). Influence of glycemic control on the levels of subgingival periodontal pathogens in patients with generalized chronic periodontitis and type 2 diabetes.. J. Appl. Oral Sci..

[46] Carelli M., Maguolo A., Zusi C., Olivieri F., Emiliani F., De Grandi G., Unali I., Zerman N., Signoretto C., Maffeis C. (2023). Oral microbiota in children and adolescents with type 1 diabetes mellitus: Novel insights into the pathogenesis of dental and periodontal disease.. Microorganisms.

[47] Negrini T.C., Carlos I.Z., Duque C., Caiaffa K.S., Arthur R.A. (2021). Interplay among the oral microbiome, oral cavity conditions, the host immune response, diabetes mellitus, and its associated-risk factors—An overview.. Front. Oral Health.

[48] Belkina A.C., Azer M., Lee J.J., Elgaali H.H., Pihl R., Cleveland M., Carr J., Kim S., Habib C., Hasturk H., Snyder-Cappione J.E., Nikolajczyk B.S. (2020). Single-cell analysis of the periodontal immune niche in type 2 diabetes.. J. Dent. Res..

[49] Almubarak A., Tanagala K.K.K., Papapanou P.N., Lalla E., Momen-Heravi F. (2020). Disruption of monocyte and macrophage homeostasis in periodontitis.. Front. Immunol..

[50] Jin L., Deng Z., Zhang J., Yang C., Liu J., Han W., Ye P., Si Y., Chen G. (2019). Mesenchymal stem cells promote type 2 macrophage polarization to ameliorate the myocardial injury caused by diabetic cardiomyopathy.. J. Transl. Med..

[51] Ackerman J.E., Geary M.B., Orner C.A., Bawany F., Loiselle A.E. (2017). Obesity/Type II diabetes alters macrophage polarization resulting in a fibrotic tendon healing response.. PLoS One.

[52] Kajiya M., Kurihara H. (2021). Molecular mechanisms of periodontal disease.. Int. J. Mol. Sci..

[53] Yang S., Li Y., Liu C., Wu Y., Wan Z., Shen D. (2022). Pathogenesis and treatment of wound healing in patients with diabetes after tooth extraction.. Front. Endocrinol..

[54] Shen X., Shen X., Li B., Zhu W., Fu Y., Xu R., Du Y., Cheng J., Jiang H. (2021). Abnormal macrophage polarization impedes the healing of diabetes-associated tooth sockets.. Bone.

[55] Maftei G.A., Martu M.A., Martu M.C., Popescu D., Surlin P., Tatarciuc D., Popa C., Foia L.G. (2021). Correlations between salivary immuno-biochemical markers and hba1c in type 2 diabetes subjects before and after dental extraction.. Antioxidants.

[56] Zgutka K., Tkacz M., Tomasiak P., Tarnowski M. (2023). A role for advanced glycation end products in molecular ageing.. Int. J. Mol. Sci..

[57] Shen C.Y., Lu C.H., Wu C.H., Li K.J., Kuo Y.M., Hsieh S.C., Yu C.L. (2020). The development of maillard reaction, and advanced glycation end product (AGE)-Receptor for AGE (RAGE) signaling inhibitors as novel therapeutic strategies for patients with AGE-related diseases.. Molecules.

[58] Taguchi K., Fukami K. (2023). RAGE signaling regulates the progression of diabetic complications.. Front. Pharmacol..

[59] Hendrijantini N., Sitalaksmi R.M., Ari M.D.A., Hidayat T.J., Putri P.A.N., Sukandar D. (2020). The expression of TNF-Î±, IL-1Î², and IL-10 in the diabetes mellitus condition induced by the combination of spirulina and chitosan.. Bali Medical J..

[60] Camargo G.A.C.G., Rodrigues J.M.S., Amorim C.S., Wenderoscky L.F., Pascoal V.D.B., Souza A.C.A., Duque C. (2022). Gene expression of inflammatory immune factors and clinical parameters in diabetes and nondiabetes patients with periodontal disease.. Res. Soc. Dev..

[61] Techatanawat S., Surarit R., Chairatvit K., Khovidhunkit W., Roytrakul S., Thanakun S., Kobayashi H., Khovidhunkit S.P., Izumi Y. (2020). Salivary and serum interleukin-17A and interleukin-18 levels in patients with type 2 diabetes mellitus with and without periodontitis.. PLoS One.

[62] Javed F, Ahmed A (2013). Proinflammatory cytokines in the saliva, gingival crevicular fluid and serum of diabetic patients with periodontal disease.. J. Res. Pract. Dent..

[63] Al Shehhi Y.I., Elemam N.M., Alsaegh M.A. (2024). The response of salivary proinflammatory biomarkers to tooth extraction in individuals with type II diabetes mellitus.. BMC Oral Health.

[64] Freitas M.O., Fonseca A.P.R., de Aguiar M.T., Dias C.C., Avelar R.L., Sousa F.B., Alves A.P.N.N., de Barros Silva P.G. (2022). Tumor necrosis factor alpha (TNF-α) blockage reduces acute inflammation and delayed wound healing in oral ulcer of rats.. Inflammopharmacology.

[65] Gazal G. (2020). Management of an emergency tooth extraction in diabetic patients on the dental chair.. Saudi Dent. J..

[66] Ko K., Sculean A., Graves D.T. (2021). Diabetic wound healing in soft and hard oral tissues.. Transl. Res..

[67] Zheng D., Liwinski T., Elinav E. (2020). Inflammasome activation and regulation: Toward a better understanding of complex mechanisms.. Cell Discov..

[68] Makaremi S., Asgarzadeh A., Kianfar H., Mohammadnia A., Asghariazar V., Safarzadeh E. (2022). The role of IL-1 family of cytokines and receptors in pathogenesis of COVID-19.. Inflamm. Res..

[69] Dai W., Dong Y., Han T., Wang J., Gao B., Guo H., Xu F., Li J., Ma Y. (2022). Microenvironmental cue-regulated exosomes as therapeutic strategies for improving chronic wound healing.. NPG Asia Mater..

[70] Nirenjen S., Narayanan J., Tamilanban T., Subramaniyan V., Chitra V., Fuloria N.K., Wong L.S., Ramachawolran G., Sekar M., Gupta G., Fuloria S., Chinni S.V., Selvaraj S. (2023). Exploring the contribution of pro-inflammatory cytokines to impaired wound healing in diabetes.. Front. Immunol..

[71] Johnson B.Z., Stevenson A.W., Prêle C.M., Fear M.W., Wood F.M. (2020). The role of IL-6 in skin fibrosis and cutaneous wound healing.. Biomedicines.

[72] Khairul-Anwar I., Wan-Nazatul-Shima S., Siti-Lailatul-Akmar Z., Siti-Azrin A.H., Zunaina E. (2022). Evaluation of TNF-α and IL-6 in saliva among diabetic retinopathy patients in East Coast Malaysia.. Trop. Med. Int. Health.

[73] Alkhashab M.M., Abdalfattah D.M., Al-Rawee R.Y. (2020). Metronidazole gel foam influence on salivary tumor necrosis factor and interleakin-6 with lower third Molar extraction (Randomized Control Clinical Study).. Oral Health Dent. Sci..

[74] Bashir H., Ahmad Bhat S., Majid S., Hamid R., Koul R., U Rehman M., Din I., Ahmad Bhat J., Qadir J., Masood A. (2020). Role of inflammatory mediators (TNF-α, IL-6, CRP), biochemical and hematological parameters in type 2 diabetes mellitus patients of Kashmir, India.. Med. J. Islam. Repub. Iran.

[75] Ringleb M., Javelle F., Haunhorst S. (2023). Acute resistance exercise-induced changes in IL-6, IL-10, and IL-1ra in healthy adults: A systematic review and meta-analysis.. medRxiv.

[76] Mojtahedi H., Hossein-Khannazer N., Mahmoud Hashemi S., Masoudnia M., Askarzadeh M., Khojasteh A., Sattari M. (2022). Effects of lipopolysaccharide from *Porphyromonas gingivalis* and *Escherichia coli* on gene expression levels of toll-like receptors and inflammatory cytokines in human dental pulp stem cells.. Iran. J. Immunol..

[77] Hutor N.S., Pidruchna S.R., Melnyk N.A. (2020). The role of prooxidant-antioxidant system in the development of alveolitis after teeth extraction.. J. Int. Dent. Med. Res..

[78] Rohmaniar P.D., Rahayu R.P., Narmada I.B. (2024). Effect of diabetes mellitus on each phase of tooth extraction socket healing.. J. Int. Dent. Med. Res..

[79] Li W., Ling W., Teng X., Quan C., Cai S., Hu S. (2016). Effect of advanced glycation end products, extracellular matrix metalloproteinase inducer and matrix metalloproteinases on type-I collagen metabolism.. Biomed. Rep..

[80] Lee J., Yun J.S., Ko S.H. (2022). Advanced glycation end products and their effect on vascular complications in type 2 diabetes mellitus.. Nutrients.

[81] Fathi S., Borzouei S., Goodarzi M.T., Poorolajal J., Ahmadi- Motamayel F. (2020). Evaluation of salivary antioxidants and oxidative stress markers in type 2 diabetes mellitus: A retrospective cohort study.. Endocr. Metab. Immune Disord. Drug Targets.

[82] Younis W.H., Al-Rawi N.H., Mohamed M.A.H., Yaseen N.Y. (2013). Molecular events on tooth socket healing in diabetic rabbits.. Br. J. Oral Maxillofac. Surg..

[83] Wagner D., Fahrleitner-Pammer A. (2010). Levels of osteoprotegerin (OPG) and receptor activator for nuclear factor kappa B ligand (RANKL) in serum: Are they of any help?. Wien. Med. Wochenschr..

[84] Buckley K.A., Fraser W.D. (2002). Receptor activator for nuclear factor kappaB ligand and osteoprotegerin: Regulators of bone physiology and immune responses/potential therapeutic agents and biochemical markers.. Ann. Clin. Biochem..

[85] Simonet W.S., Lacey D.L., Dunstan C.R., Kelley M., Chang M.S., Lüthy R., Nguyen H.Q., Wooden S., Bennett L., Boone T., Shimamoto G., DeRose M., Elliott R., Colombero A., Tan H.L., Trail G., Sullivan J., Davy E., Bucay N., Renshaw-Gegg L., Hughes T.M., Hill D., Pattison W., Campbell P., Sander S., Van G., Tarpley J., Derby P., Lee R., Boyle W.J. (1997). Osteoprotegerin: A novel secreted protein involved in the regulation of bone density.. Cell.

[86] Anderson D.M., Maraskovsky E., Billingsley W.L., Dougall W.C., Tometsko M.E., Roux E.R., Teepe M.C., DuBose R.F., Cosman D., Galibert L. (1997). A homologue of the TNF receptor and its ligand enhance T-cell growth and dendritic-cell function.. Nature.

[87] Fukushima H., Kajiya H., Takada K., Okamoto F., Okabe K. (2003). Expression and role of RANKL in periodontal ligament cells during physiological root-resorption in human deciduous teeth.. Eur. J. Oral Sci..

[88] Lossdörfer S., Götz W., Jäger A. (2002). Immunohistochemical localization of receptor activator of nuclear factor kappaB (RANK) and its ligand (RANKL) in human deciduous teeth.. Calcif. Tissue Int..

[89] Kumamoto H., Ooya K. (2004). Expression of parathyroid hormone-related protein (PTHrP), osteoclast differentiation factor (ODF)/receptor activator of nuclear factor-κB ligand (RANKL) and osteoclastogenesis inhibitory factor (OCIF)/osteoprotegerin (OPG) in ameloblastomas.. J. Oral Pathol. Med..

[90] Lacey D.L., Timms E., Tan H.L., Kelley M.J., Dunstan C.R., Burgess T., Elliott R., Colombero A., Elliott G., Scully S., Hsu H., Sullivan J., Hawkins N., Davy E., Capparelli C., Eli A., Qian Y.X., Kaufman S., Sarosi I., Shalhoub V., Senaldi G., Guo J., Delaney J., Boyle W.J. (1998). Osteoprotegerin ligand is a cytokine that regulates osteoclast differentiation and activation.. Cell.

[91] Aubin J.E., Bonnelye E. (2000). Osteoprotegerin and its ligand: A new paradigm for regulation of osteoclastogenesis and bone resorption.. Osteoporos. Int..

[92] Yu H., de Vos P., Ren Y. (2011). Overexpression of osteoprotegerin promotes preosteoblast differentiation to mature osteoblasts.. Angle Orthod..

[93] Udagawa N., Takahashi N., Yasuda H., Mizuno A., Itoh K., Ueno Y., Shinki T., Gillespie M.T., Martin T.J., Higashio K., Suda T. (2000). Osteoprotegerin produced by osteoblasts is an important regulator in osteoclast development and function.. Endocrinology.

[94] Santos V.R., Lima J.A., Gonçalves T.E.D., Bastos M.F., Figueiredo L.C., Shibli J.A., Duarte P.M. (2010). Receptor activator of nuclear factor-kappa B ligand/osteoprotegerin ratio in sites of chronic periodontitis of subjects with poorly and well-controlled type 2 diabetes.. J. Periodontol..

[95] Cirelli T., Nicchio I.G., Bussaneli D.G., Silva B.R., Nepomuceno R., Orrico S.R.P., Cirelli J.A., Theodoro L.H., Barros S.P., Scarel-Caminaga R.M. (2023). Evidence linking *PPARG* genetic variants with periodontitis and type 2 diabetes mellitus in a Brazilian population.. Int. J. Mol. Sci..

[96] Wei X., Zhang X., Chen R., Li Y., Yang Y., Deng K., Cai Z., Lai H., Shi J. (2024). Impact of periodontitis on type 2 diabetes: A bioinformatic analysis.. BMC Oral Health.

[97] Cao X., Huo P., Li W., Li P., He L., Meng H. (2019). Interactions among moderate/severe periodontitis, ADIPOQ-rs1501299, and LEPR-rs1137100 polymorphisms on the risk of type 2 diabetes in a Chinese population.. Arch. Oral Biol..

[98] Wu K.C.H., Liu L., Xu A., Chan Y.H., Cheung B.M.Y. (2024). Shared genetic architecture between periodontal disease and type 2 diabetes: A large scale genome-wide cross-trait analysis.. Endocrine.

[99] López N.J., Valenzuela C.Y., Jara L. (2009). Interleukin-1 gene cluster polymorphisms associated with periodontal disease in type 2 diabetes.. J. Periodontol..

[100] Silva A.N.A., Nicchio I.G., da Silva B.R., Martelli M.G.G., Hidalgo M.A.R., Nepomuceno R., Theodoro L.H., Cirelli J.A., Orrico S.R.P., Cirelli T., Barros S.P., Scarel-Caminaga R.M. (2022). Polymorphisms in risk genes of type 2 diabetes mellitus could be also markers of susceptibility to periodontitis.. Arch. Oral Biol..

[101] Chen H., Peng L., Wang Z., He Y., Tang S., Zhang X. (2022). Exploration of cross-talk and pyroptosis-related gene signatures and molecular mechanisms between periodontitis and diabetes mellitus *via* peripheral blood mononuclear cell microarray data analysis.. Cytokine.

[102] Kang J., Kwon E.J., Ha M., Lee H., Yu Y., Kang J.W., Kim Y., Lee E.Y., Joo J.Y., Heo H.J., Kim E.K., Kim T.W., Kim Y.H., Park H.R. (2022). Identification of shared genes and pathways in periodontitis and type 2 diabetes by bioinformatics analysis.. Front. Endocrinol..

[103] Pan S., Hu B., Sun J., Yang Z., Yu W., He Z., Gao X., Song J. (2022). Identification of cross-talk pathways and ferroptosis-related genes in periodontitis and type 2 diabetes mellitus by bioinformatics analysis and experimental validation.. Front. Immunol..

[104] Li Y., Du Z., Xie X., Zhang Y., Liu H., Zhou Z., Zhao J., Lee R.S.B., Xiao Y., Ivanoviski S., Yan F. (2021). Epigenetic changes caused by diabetes and their potential role in the development of periodontitis.. J. Diabetes Investig..

[105] Kang J., Lee H., Joo J.Y., Song J.M., Kim H.J., Kim Y.H., Park H.R. (2024). Comparison of genetic and epigenetic profiles of periodontitis according to the presence of type 2 diabetes.. MedComm.

[106] Barros S.P., Hefni E., Nepomuceno R., Offenbacher S., North K. (2018). Targeting epigenetic mechanisms in periodontal diseases.. Periodontol. 2000.

[107] Lontchi-Yimagou E., Sobngwi E., Matsha T.E., Kengne A.P. (2013). Diabetes mellitus and inflammation.. Curr. Diab. Rep..

[108] Hanes P.J., Krishna R. (2010). Characteristics of inflammation common to both diabetes and periodontitis: Are predictive diagnosis and targeted preventive measures possible?. EPMA J..

[109] Chopra A., Jayasinghe T.N., Eberhard J. (2022). Are inflamed periodontal tissues endogenous source of advanced glycation end-products (AGEs) in individuals with and without diabetes mellitus? A systematic review.. Biomolecules.

[110] Silva D.N.A., Casarin M., Monajemzadeh S., Bezerra B.B., Lux R., Pirih F.Q. (2022). The microbiome in periodontitis and diabetes.. Front. Oral Health.

[111] Brunkwall L., Orho-Melander M. (2017). The gut microbiome as a target for prevention and treatment of hyperglycaemia in type 2 diabetes: From current human evidence to future possibilities.. Diabetologia.

[112] Shivanand S., Savita S., Rithesh K., Singh N. (2016). Nutrigenomics: A new paradigm for revealing periodontal inter-relationships.. J. Biomed. Pharm. Res..

[113] Ramos-Lopez O., Milagro F.I., Riezu-Boj J.I., Martinez J.A. (2021). Epigenetic signatures underlying inflammation: An interplay of nutrition, physical activity, metabolic diseases, and environmental factors for personalized nutrition.. Inflamm. Res..

